# Modern Osteopathy

**Published:** 1911-07-22

**Authors:** 


### SPECIAL ARTICLE.

### MODERN OSTEOPATHY.

BY A "MEDICAL OSTEOPATH."

The term "Osteopathy" conveys little or no meaning to the majority of English readers, both medical and lay, but in America this system of therapeutics is becoming increasingly adopted, not only by those who have suffered "many things of many physicians and rather grown worse," but also by those threatened with more acute conditions, who welcome any therapeutic measures that appeal to their judgment as rational or natural.

Some forty years ago Dr. Andrew Taylor Still, a medical officer of the U.S. Army, a veteran of the Civil War, many of whose relations were of the medical profession, saw three of his children die of meningitis, in spite of all that he or his medical confrères could do. When the fourth was threatened with a similar fate, it occurred to him to use manipulative treatment to the neck and spine, and the child recovered.

This was the impetus which resulted in an exhaustless special study of human anatomy and physiology, and eventuated in the formulation of a definite system of manipulative therapeutics, named by him osteopathy. As a matter of experience, he found that the sub-luxation of bones was one of the most constant causes of diseased conditions, and as the bony framework is used as a basis for physical examination and for restoring misplaced parts of the body, the name osteopathy was adopted. The term has been regarded by some as misleading because it emphasises a *part* only of this system, which deals equally with impediments in the nature of contractured muscles, ruptured ligaments, constricted or dilated vessels, hypertrophied, or congested conditions of tissue, and hence the term "manual or manipulative therapeutics" is considered preferable by many.

As a definition the following seems to be clear and comprehensive. "Osteopathy is a system of medicine characterised by close adherence to the physiological axiom that perfect health depends on a perfect circulation and perfect nerve control in every tissue of the body. Its ætiology emphasises physical perversions of tissue relations as causes of disease. Its diagnosis is mainly dependent on the discovery of physical lesions, by means of palpation. Its therapeutics comprehends (1) manipulation (including surgery) for purposes of readjusting tissue relations; (2) scientific dietetics; (3) personal and public hygiene."—(D. L. Tasker, President of Medical State Board of California.)

The rapid growth of the movement is shown by the fact that within fifteen years eight colleges have been established, and there are now more than 5,000 osteopathic physicians practising in the United States and other countries. Graduate osteopaths receive legal recognition either by direct legislation or judicial decision in thirty-seven States of the Union, and are employed by one hundred insurance companies and by many railway and other corporations in the settlement of personal injury cases. The course of study extends over three years, and in some colleges is being extended to four years, the subjects being much the same as in a medical school, with the exception of materia medica and major surgery. The osteopathic conception is that the body contains all the forces necessary for its conservation and repair; hence the treatment limits itself to the removal of all obstructions to those forces and deprecates the administration of vegetable or mineral poisons, which, not being capable of assimilation as food, must perforce be thrown out of the body by an expenditure of energy, dearly bought from an already enfeebled system.

It is a matter of common knowledge that the greater the physician the rarer and shorter his prescription; in fact, many openly admit that they "give medicine because the patient expects it," *i.e.* really as a form of suggestion. If there is any truth in the following statements by eminent physicians that drug-giving at best "is putting something of which we know little into a body of which we know less," that "mankind has been drugged to death," that "the effects of medicine on the human system are in the highest degree uncertain, except that they have destroyed more lives than war, pestilence, and famine combined"; then surely a system, working on a scientific basis, producing results that compare favourably with those of other systems, deserves to be received with toleration and candour.

Much has been said and written lately about the "Hinterland of Surgery," about scientific and unscientific bone-setting and irregular practice, but, after all, every reasonable person has a right to take the shortest cut to cure or relief. If he finds that his family doctor is too stereotyped, too much of an adherent to tradition, or a devotee of the "expectant" treatment, may he not naturally be drawn to tread even a new and untried path, if it offers any substantial prospect of speedy relief and possible cure. Has there not been in the past a good deal of false pride, which would regard as derogatory any manipulation short of surgery, as if the minor forms of bloodless surgery, the practical *seeing with the hands*, and rectifying a condition without an exploratory incision, were not as honourable as a diagnosis on operation, or the writing of a prescription, without knowledge of a patient's idiosyncrasies and power of reaction?

If the public confidence in the medical profession is to be upheld, surely *every* form of therapeutics should be welcomed for investigation, and, when proved of value, incorporated into the physician's armentarium. Every practitioner cannot have a specialised knowledge of every known means of cure, but it is his business to discriminate as to the best treatment for each individual who consults him, and if he cannot administer it himself to put his patient in the way of obtaining it.

Although the late Dr. Wharton Hood had a full medical training and degree, yet, because he combined with it a knowledge of the much derided and belittled "bone-setting" (which knowledge he was willing to impart to his medical brethren), he was looked upon by many as an "irregular practitioner." Must the suffering laity be deterred from seeking the relief and cure which a good osteopath can give in many instances, until the medical profession has decided whether such treatment should be recognised as ethical and absolutely professional?

In America, where the population is largely a survival of the fittest of many nations, many medical men and women have taken this course in manipulative therapeutics, and are thus qualified to treat either by medication or manipulation, and some from this country have also attended lectures. One recent writer mentions several cases which he treated very successfully on the limited knowledge thus gained, and yet makes the somewhat contradictory assertion that hydrotherapy, massage, electricity, etc., are of more value; this being no doubt due to habitual deference to that tyrant, professional etiquette. It seems a pity that this European form of the "caste" spirit should prevent the medical profession from giving a fair hearing to the latest and most scientific form of manual therapeutics, combining as it does apparently all that was valuable in the systems that preceded it.

It is especially noticeable that certain diseases which have been regarded as more or less chronic and incurable in character and have defied other methods, often yield quickly to osteopathic treatment, *e.g.* neuritis, rheumatism, neuralgia, constipation.

As harmony is one of the leading laws of life, the essential treatment of disease is surely to seek out deviations from the normal in each and every tissue and readjust as far as may be all physical and mental kinks, till the whole human machine works up to the highest standard possible for that individual.

